# Extraction procedures for the study of phytotoxicity and degradation processes of selected triketones in a water ecosystem

**DOI:** 10.1007/s11356-013-2425-z

**Published:** 2013-12-21

**Authors:** Hanna Barchanska, Anna Kowalska, Barbara Poloczek

**Affiliations:** Department of Inorganic, Analytical Chemistry and Electrochemistry, Faculty of Chemistry, Silesian University of Technology, B. Krzywoustego 6 Str, 44-100, Gliwice, Poland

**Keywords:** Triketone herbicides, *Egeria densa*, Chlorophyll, Matrix solid-phase dispersion, Solid-phase extraction, Risk assessment

## Abstract

Simple and effective extraction methods based on matrix solid-phase dispersion (MSPD), dispersive liquid–liquid microextraction (DLLME), and solid-phase extraction (SPE) coupled with high-performance liquid chromatography with diode array detector (HPLC-DAD) were developed to determine triketone herbicides—sulcotrione (SUL), mesotrione (MES), tembotrione (TEMB), and their degradation products—in plant tissues and water samples. The extraction procedures were employed to enable quantification of the accumulation of selected triketone herbicides and their degradation products in a model aquatic plant, *Egeria densa*. To obtain comprehensive information about the triketones' influence on an aquatic plant, changes in chlorophyll concentration in plants exposed to these triketones were monitored. The average recovery ranged from 58 to 115 % (coefficients of variation 7–12 %) for plant tissues and from 52 to 96 % (coefficients of variation 8–20 %) for water samples. The limit of detection (LOD) for the MSPD–HPLC-DAD procedure was in the range of 0.06–0.23 μg/g, whereas for DLLME–HPLC-DAD and SPE–HPLC-DAD, LOD was in the range of 0.06–0.26 μg/mL. Symptoms of the phytotoxicity of sulcotrione, mesotrione, tembotrione, and their degradation products (decrease of chlorophyll concentration in plant sprouts) were observed for *E. densa* cultivated in water with herbicide concentrations of 100 μg/L. Moreover, the tembotrione degradation product exhibited a high level of accumulation and low metabolism in plant tissues in comparison to the other triketones and their degradation products.

**Electronic supplementary material:**

The online version of this article (doi:10.1007/s11356-013-2425-z) contains supplementary material, which is available to authorized users.

## Introduction

In a changing environment, plants are exposed to environmental stressors such as pesticides. Triketone herbicides were registered in European Union countries at the beginning of the twenty-first century (Batisson et al. 2009). They are used for pre- and post-emergence weed control, mainly in maize cultivation. These compounds inhibit *p*-hydroxyphenylpyruvate dioxygenase (HPPD), an enzyme responsible for catalyzing the conversion of tyrosine to plastoquinone and α-tocopherol (Soltani et al. 2011). The inhibition of this enzyme disrupts the biosynthesis of carotenoids, resulting in foliage bleaching as an effect of the chlorophyll loss. The most frequently used triketones are mesotrione (MES), sulcotrione (SUL), and tembotrione (TEMB). The systematic names and the structures, as well as selected physical–chemical properties, of these compounds and their degradation products are presented in Table 1.

**Table 1:**
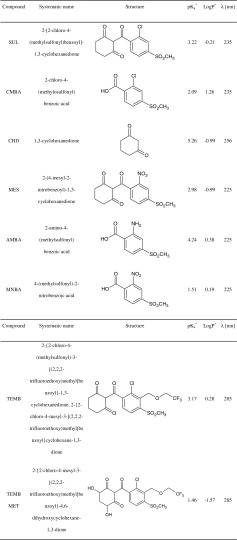
Characteristic of analytes

^a^Octanol–water partition constants and ionization constant were obtained from ACD/Labs (SciFinder)

After application on agricultural areas, pesticides may enter freshwater ecosystems due to a combination of spray, drift, runoff, atmospheric deposition, and spillages (Knauer and Hommen 2013).

The United States Environmental Protection Agency (US-EPA) revealed that MES is unstable in the environment and decomposes into two degradation products: 4-(methylsulfonyl)-2-nitrobenzoic acid (MNBA) and 2-amino-4-(methylsulfonyl) benzoic acid (AMBA) (US-EPA 2001), which are more toxic than the parent compound (Bonnet at al. 2008).

In water, SUL is stable at pH values between 4 and 9. Below this range, 2-chloro-4-(methylosulfonyl)benzoic acid (CMBA) and 1,3-cyclohexanedione (CHD) are formed (Chaabane et al. 2007). The degradation path of tembotrione is not well elucidated.

Although some toxicity data already exists for pharmaceuticals and pesticides toward selected bacteria, macrophytes, fungi, and crops (Hanson et al. 2006; Chaabane et al. 2007; Soltani et al. 2011; Knauer and Hommen 2013), knowledge of the ecological effects of triketones and their degradation products is generally lacking. Regardless of the existing research into the toxicity of pharmaceuticals and pesticides in the case of aquatic plants, there is a lack of data concerning the influence of triketone herbicides and their degradation products.

Triketone herbicides, along with their degradation products, are frequently found in the aquatic environment. Therefore, to understand their impact on aquatic food webs, it is necessary to determine their bioaccumulation in aquatic plant tissues.

To determine the influence of herbicides on aquatic plants, the determination of their accumulation level in vegetation tissues is insufficient. In plants, fluctuations of many chemical compounds such as chlorophyll (Casati et al. 2002; Ferrat et al. 2003; Hanson et al. 2006; Riis et al. 2012; Su et al. 2012), soluble proteins, soluble carbohydrates, malondialdehyde, and antioxidant enzymes (Casati et al. 2002; Malec et al. 2009; Su et al. 2012) are important indexes to measure the response of physiological stress. Triketones disturb the photosynthesis process, leading to a decrease in chlorophyll concentration in plants. Therefore, in the present research regarding the risk assessment of triketones, changes of chlorophyll content in plant tissues exposed to these herbicides were chosen.

*Egeria densa*, commonly known as Brazilian waterweed, is a widely used plant in the ornamental fish industry via aquarium trades. It lives in an environment with moderately high light intensity and conducts photosynthesis in stems and leaves. It is an introduced invasive species and, in moderate climate regions, is found as a nuisance species (Dillon et al. 1988; Hanson et al. 2006; Csurhes et al. 2008; Marin et al. 2009; Reid et al. 2009; Su et al. 2012). Because of the ease in propagation through the double node, root crown, or apical shoot, *E. densa* has been used as a model plant to assess water quality and heavy metal accumulation and toxicity, as well as the metabolism of pesticides and pharmaceuticals in plants (Ferrat et al. 2003; Hanson et al. 2006; Malec et al. 2009; Favas et al. 2012; Riis et al. 2012; Su et al. 2012).

The objective of the reported work was the determination of the influence of selected triketone herbicides (SUL, MES, and TEMB, as well as their degradation products) on the aquatic plant *E. densa* in the microcosm. To achieve this aim:

1. Matrix solid-phase dispersion (MSPD) was applied for the abovementioned analytes extracted from plant tissues. This extraction technique is flexible, versatile, rapid, and low cost. Furthermore, it requires a small sample aliquot and provides the extraction and cleanup in a single step (Barker 2007; Rodrigues et al. 2010; Jin et al. 2012; Zhang et al. 2012; Capriotti et al. 2013). Dispersive liquid–liquid microextraction (DLLME) and solid-phase extraction (SPE) were employed for the extraction of analytes from water samples. Both techniques require low consumption of solvents and have high analyte recoveries. All extraction techniques were coupled to high-performance liquid chromatography with diode array detector (HPLC-DAD). These procedures enabled us to monitor herbicide degradation processes in water and the herbicides' accumulation level in plant tissues.
2. Chlorophyll was chosen as a biomarker to assess the toxicity of triketone herbicides. The spectrophotometric analytical procedure for chlorophyll determination in plant tissues was applied according to Su et al. (2010).
3. The correlation of the triketone concentrations with the chlorophyll content in plant tissues was performed. The obtained data were applied for a preliminary risk assessment for *E. densa* with triketone herbicides.

## Materials and methods

### Chemicals and apparatus

All chemical standards were of an analytical reagent grade. Standards of herbicides and their degradation products were supplied by Sigma-Aldrich, Germany (MES, SUL, TEMB, and TEMB MET); Santa Cruz Biotechnology, Germany (AMBA and MNBA); and Dr. Ehrenstorfer Quality, Germany (CHD and CMBA). Stock standard solutions for each compound were prepared in methanol at 1 mg/mL and stored in glass vials at 4 °C in the dark. Acetonitrile, trifluoroacetic acid (TFA), and water (all HPLC grade) were from Merck, Germany. Acetone, acetonitrile, carbon tetrachloride, chloroform, formic acid, and methanol (all analytical grade) were purchased from POCH, Poland. FLORAMIX was supplied by INTERMAG, Poland. Silica gel, cellulose, and aluminum oxide were provided by Merck, Germany.

The chromatographic system used (Merck Hitachi, Germany) consisted of a Hitachi LaChrom Elite L-2130 pump and Hitachi LaChrom Elite L-2455 photodiode array detector (DAD). The stationary-phase column was LiChroCART Purospher RP-18e (125 × 3 mm, 5 μm, Merck). The mobile phase consisted of 0.05 % TFA in water (A) and acetonitrile (B). The gradient profile during separation was as follows: 0 min: 100 % A, flow: 0.7 mL/min; 28 min: 60 % A, 40 % B, flow: 0.7 mL/min; 35 min: 30 % A, 70 % B, flow: 1.0 mL/min. Analytical wavelengths for each compound (Table 1) were used for the quantitative analysis. A spectrophotometer (Pye Unicam, UK) was used for chlorophyll determination. Solid-phase extraction was performed using a J.T. Baker (Holland) vacuum manifold.

Solid-phase extraction cartridge columns were obtained from Agilent, Germany (silica gel modified with octadecyl chains (Bond Elut ENV), 500 mg, 6 mL); J.T. Baker, Holland (styrene–divinylbenzene (SDB), 500 mg, 6 mL); and Waters, USA (copolymer styrene–*N*-vinylpyrrolidone (Oasis HLB), 500 mg, 6 mL). Membranes (0.20-μm pore size) were purchased from J.T. Baker, Holland.

### Experimental design

Waterweed was bought in an aquarium shop. In the laboratory, *E. densa* was cultivated in nine glass containers with lids (four shoots, about 15 cm long per container, three replicates) filled with 2 L of distilled water with a rooting substrate (FLORAMIX, 2 mL/10 L). Plants were grown in a day/night cycle of 16:8 h and a temperature regime of 25–20 °C. After 3 weeks of acclimation, to each of the seven containers, a single herbicide standard solution was added. To estimate the phytotoxicity concentration of the triketones, the plant was cultivated at the following concentration levels of each analyte: 10, 50, and 100 μg/L. In parallel, *E. densa* sprouts were cultivated in pure water (the eighth container; blank sample). Every 7 days after herbicide application, the concentration of triketones and their degradation products, as well as changes of chlorophyll content in plant tissues, was determined. At the same time, the concentration of herbicides in water where plants were cultivated was monitored. The experiment was carried out for a period of 4 weeks. For analyses, 0.5 cm of each shoot in a given container was cut, mixed, and homogenized. Subsequently, 0.5 and 0.1 g of such prepared plant tissues were used for the MSPD procedure and chlorophyll determination. Each analysis was conducted in triplicate.

#### MSPD for the extraction of triketones from plant tissues

**Fig. 1:**
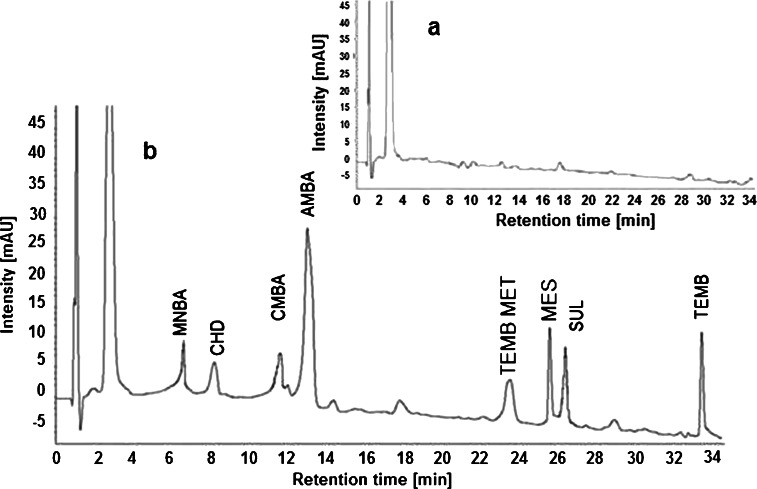
Chromatogram of the blank plant extract (**a**) and spiked plant extract (**b**) (λ = 225 nm) obtained by the MSPD (Al_2_O_3_/1 % HCOOH in CH_3_OH)–HPLC-DAD procedure

About 0.5 g of representative portions of plant tissues was homogenized with 1.0 g of aluminum oxide in a porcelain mortar. The homogeneous mixture was transferred into a PE cartridge with a sinter underlying. Another sinter was placed on top of the sample–sorbent mixture. The sample mixture was gently pressed and the cartridge was connected to a SPE vacuum manifold. A solvent (1 % HCOOH in methanol) was then introduced to elute the herbicides from the cartridge and the eluent was collected. Before chromatographic analysis, the solvent was evaporated and the dry residue was dissolved in 1 mL of methanol. A description of extraction condition optimization is detailed within the Supplementary Material. The linearity of the MSPD–HPLC-DAD method was tested with standard mixtures at six concentration levels (*n* = 3) in the range of 0.2–7.0 μg/mL. The recoveries of analytes were in the range of 58–115 %. The extracts obtained according to this procedure did not contain matrix compounds that disturb the chromatographic analysis, as illustrated in Fig. 1.

#### Determination of chlorophyll in plant tissues

Determination of chlorophyll was conducted on the basis of the research described by Su et al. (2010). About 0.1 g (*W*) of homogenized *E. densa* tissues and a small amount of CaCO_3_ were mixed with 90 % acetone in water. Next, the mixture was filtered through the sinter and diluted with 90 % acetone in water up to 25 mL (*V*) in a measuring flask. Without any delay, the absorbance was measured by means of a spectrophotometer. The measurements were conducted at two wavelengths (645 and 663 nm). The concentration of chlorophyll was calculated on the basis of the following equations:

1 $$ C\left(\upmu \mathrm{g}/\mathrm{mL}\right)=20.31{A}_{645}+8.05{A}_{663} $$

2 $$ C\left(\mathrm{mg}/\mathrm{g}\right)=\frac{C\cdot V}{W\cdot 1,000} $$

where *C* is the concentration of chlorophyll, *A* is the absorbance, *V* is the final sample volume (mL), and *W* is the mass of the sample (g).

#### DLLME for the extraction of triketones from water samples

A detailed investigation into the selection of DLLME conditions was carried out. The accurate description of DLLME optimization is placed in the Supplementary Material. For the determination of triketones and their degradation products in real water samples, the following procedure was proposed.

 About 10 mL of water sample was shaken vigorously for 2 min with 2 mL of the mixture of acetone/acetonitrile (1:1, *v*/*v*) as a dispersant solvent and 100 μL of chloroform as an extraction solvent. Next, the mixture was left for 10 min to allow the phases to separate. Finally, the lower phase was separated and evaporated to dryness under a gentle stream of nitrogen. The remaining residue was dissolved in 1.0 mL of methanol prior to HPLC analysis. The recoveries of all analytes were in the range of 81–96 %.

#### SPE for the extraction of triketones from water samples

For the verification of the results obtained by DLLME, SPE was also employed. For determination of analytes in water samples, the following procedure was used. A water sample (250 mL) was aspirated through the HLB sorbent, previously conditioned with 6 mL of methanol and subsequently 6 mL of water. Analytes were eluted by means of 6 mL of the mixture acetonitrile–methanol (1:1, *v*/*v*). After analyte elution, the extract was evaporated to dryness under a stream of nitrogen and the residue was dissolved in 1 mL of methanol. The recoveries of all analytes were in the range of 52–88 %. The calibration plot linearity of the DLLME–HPLC-DAD and SPE–HPLC-DAD methods was determined at six concentration levels (*n* = 3) in the range of 1.25–40 μg/mL in water matrices. The description of optimization of the SPE condition is placed in the Supplementary Material.

**Fig. 2:**
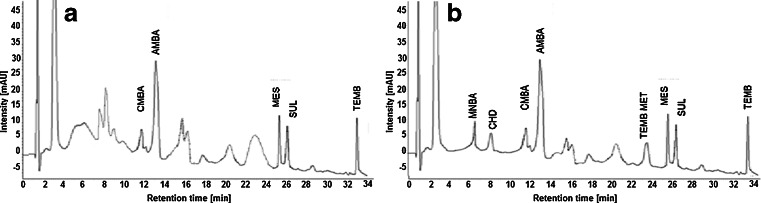
Chromatograms of spiked sewage treatment plant water obtained by DLLME (disperser: acetone/acetonitrile, extraction solvent: chloroform) (**a**) and SPE (HLB/acetonitrile/methanol) (**b**)

The prepared DLLME and SPE procedures were applied for surface waters of different origin (spring water, river water, and sewage treatment plant water). Although both extraction techniques provide high recoveries, SPE gives better elimination of matrix compounds, as illustrated in Fig. 2.

Significant matrix effects made the detection of MNBA, CHD, and TEMB MET by means of DLLME impossible. Moreover, this technique revealed higher values of CV (15–20 %) in comparison to SPE (8–12 %). Therefore, the SPE–HPLC-DAD procedure was validated and applied in the present research for triketone determination in water.

The limit of detection (LOD) was estimated as being three times the standard deviation of three replicate analyses of the standard prepared in matrix extracts at the lowest concentrations of the range of the calibration curves. The limit of quantification (LOQ) was calculated as threefold of LOD. LOD for the MSPD–HPLC-DAD procedure was in the range of 0.06–0.23 μg/g (LOQ 0.18–0.70 μg/g), whereas for SPE–HPLC-DAD, LOD was in the range of 0.06–0.26 μg/mL (LOQ 0.18–0.79 μg/mL).

Identification of the target compounds was based on the retention times, standard addition, and comparison of the UV spectra. The target compounds were quantified based on the calibration plots. The detailed parameters of calibration curves are presented in Table 2.

**Table 2:** Analytical figure of merit of the proposed methods

Analytes	SPE						MSPD					
	Linear range [μg/mL]	Slope ± s (×10^4^)	Intercept ± s (×10^4^)	*R* ^2^	LOD [μg/mL]	LOQ [μg/mL]	Linear range [μg/mL]	Slope ± s (×10^4^)	Intercept ± s (×10^3^)	*R* ^2^	LOD [μg/g]	LOQ [μg/g]
SUL	0.6–20.0	20 ± 1	27 ± 2	0.9958	0.09	0.28	0.9–7.0	19 ± 2	8 ± 1	0.9923	0.23	0.70
CMBA	0.6–20.0	9 ± 1	31 ± 4	0.9920	0.07	0.22	0.2–7.0	13 ± 2	30 ± 5.0	0.9829	0.06	0.18
CHD	0.6–20.0	81 ± 2	−24 ± 3	0.9949	0.07	0.21	0.2–0.7	72 ± 2	15 ± 1	0.9942	0.06	0.18
MES	0.6–20.0	68 ± 2	−22 ± 4	0.9961	0.11	0.33	0.4–7.0	25 ± 1	69 ± 5	0.9951	0.12	0.35
AMBA	0.6–20.0	51 ± 3	3 ± 1	0.9904	0.26	0.79	0.2–7.0	57 ± 2	263 ± 7	0.9961	0.06	0.18
MNBA	0.6–20.0	14 ± 1	7 ± 1	0.9979	0.90	0.28	0.9–7.0	5 ± 1	11 ± 1	0.9938	0.23	0.70
TEMB	0.6–20.0	15 ± 1	−8 ± 1	0.9964	0.06	0.18	0.4–7.0	17 ± 1	−2 ± 0.2	0.9903	0.12	0.35
TEMB MET	0.6–20.0	26 ± 1	32 ± 5	0.9956	0.11	0.33	0.4–7.0	19 ± 1	98 ± 2	0.9981	0.12	0.35

*s* standard deviation

## Results and discussion

The concentrations of chlorophyll as well as triketone herbicides and their degradation products in aqueous plant tissues were determined before and every 7 days after herbicide application. Simultaneously, analyte concentrations in water were determined. The experiments were conducted at different concentration levels of triketones; however, no negative effects of triketones on the growth of *E. densa* at lower concentration levels were observed. Accumulation of triketones and the decrease of chlorophyll concentration in plant tissues were observed for the herbicide concentration in water of 100 μg/L. Therefore, the discussion mentioned below concerns the results obtained for this concentration.

**Fig. 3:**
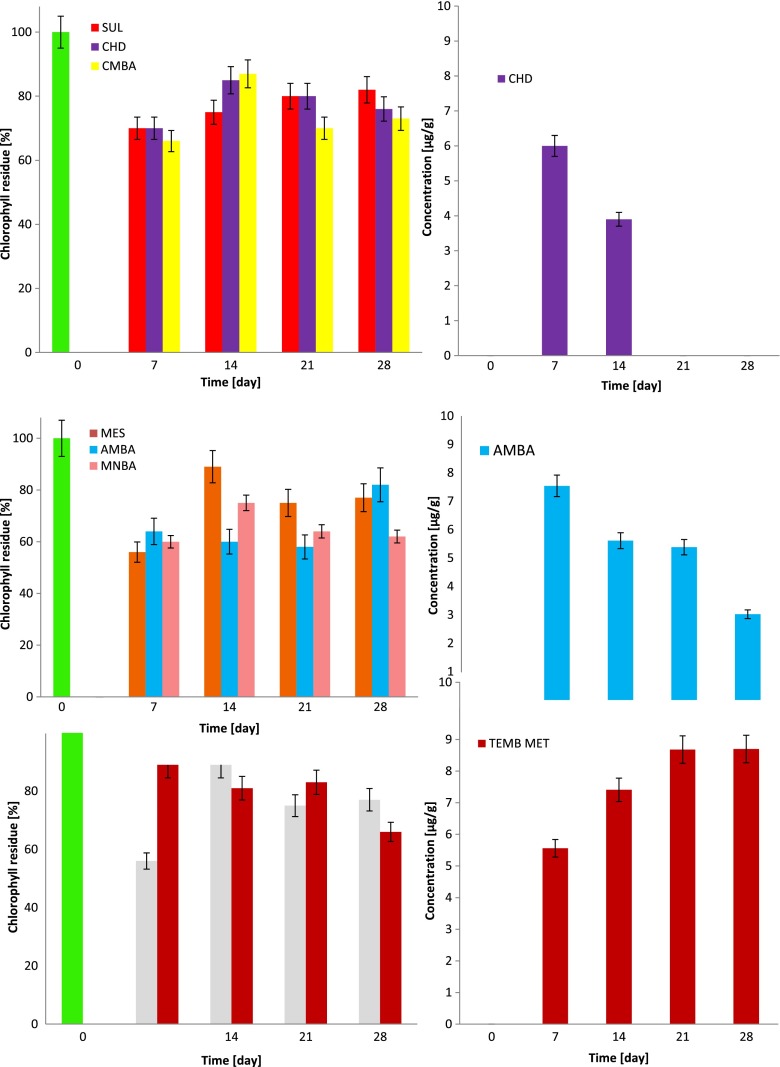
Changes of chlorophyll content and herbicide accumulation in plant tissues

The changes of chlorophyll concentration in plant tissues as a result of herbicide interaction are presented in Fig. 3.

Seven days after application of SUL and its degradation products, the chlorophyll concentration in waterweed decreased by approximately 30 % in comparison to the blank sample (first column in Fig. 3). At the same time, the changes in herbicide concentrations in water were insignificant (about 90–95 % of the initial concentration). CHD accumulation in plant tissues was also observed (6.00 μg/g).

After the next 7 days, in all cases, an increase of chlorophyll concentration was noted, with the highest increment observed in *E. densa* tissues treated with CMBA (21 % increase in comparison to the value obtained on the 7th day of the experiment). This increase was probably caused by the decrease in the concentration of sulcotrione degradation products in water. At the same time, the slope of CHD concentration to the value of 3.90 μg/g in waterweed was observed.

Between the 21st and the 28th day of the experiment, the changes of chlorophyll concentration in plant tissues were insignificant (3–6 %), which were caused by the low concentrations of CHD and CMBA in water. The analyte concentrations in water slowly decreased, reaching the 50 % level of the initial value on the 28th day of the experiment.

One week after the beginning of the experiment, a decrease of chlorophyll concentration (about 40 % of the value obtained for the blank sample) was observed in the waterweed shoots cultivated in solutions of MES and its degradation products. The accumulation of AMBA (7.54 μg/g) in plant tissues was also measured at this time. The changes of analyte concentration in water were around 5–10 % of the initial value.

In the next 20 days of the experiment, the content of chlorophyll in waterweed cultivated in AMBA solution stayed at a constant level (56–60 % of the value obtained for blank samples). During the same period, the AMBA concentration in plant tissues was measured to be 5.38–5.61 μg/g. The increase of chlorophyll in waterweed was observed until the AMBA concentration in plant tissue was as low as 3.02 μg/g (28th day of the experiment). The slope of the AMBA concentration in waterweed was caused by its degradation in water (35 % of the initial value on the 28th day of the experiment) as well as degradation processes occurring in the plant.

The influence of MES, MNBA, and TEMB on the waterweed was similar to that of SUL, except that the latter exhibited lower phytotoxicity. The degradation process of MES and TEMB in water, similar to SUL, was slow. On the 28th day of the experiment, triketone concentrations in water were around 80 % of the initial values.

Seven days after the beginning of the experiment, in the waterweed tissues cultivated in the TEMB MET solution, the decrease of chlorophyll was 10 % in comparison to the blank sample. At the same time, this analyte was detected in plant tissues at a level of 5.56 μg/g. In the following days, the decrease of chlorophyll in waterweed was proceeding (to the value of 66 % in comparison to blank sample on the 28th day of the experiment); this was accompanied by the increase of TEMB MET in plant tissues (up to the value of 8.70 μg/g). The concentration of TEMB MET in water was 59 μg/L on the 28th day of the experiment.

Among all investigated herbicides and their degradation products, the highest accumulation level was noticed for the most polar compounds: TEMB MET (log*P* = −1.57) and CHD (log*P* = −0.99).

## Conclusion

Effective and easy-to-conduct analytical procedures for simultaneous extraction and determination of selected triketone herbicides (SUL, MES, and TEMB as well as their degradation products) in aquatic plant and water matrices were developed. The MSPD–HPLC-DAD procedure, employed for plant tissues, provided satisfactory recoveries (58–115 %) and low LOD (0.06–0.23 μg/g). The proposed DLLME–HPLC-DAD protocol was characterized by recovery as being within the 72–92 % range. However, because of matrix effects, it is not recommended to extract triketone herbicides from surface water matrices.

The analytical procedures were employed for the determination of the influence of triketone herbicides on a model aquatic plant, *E. densa*. Based on this study, we conclude that triketones and their degradation products did not affect *E. densa* chlorophyll content over the course of the laboratory incubation when exposed to the selected range of concentrations. The toxicological studies on *E. densa* showed an absence of phytotoxicological symptoms up to 100 μg/L for both triketones and their degradation products. MES and its degradation products at the concentration of 100 μg/L caused the highest slope of chlorophyll concentration in plant tissues among all investigated compounds. However, according to our research, TEMB MET is the most significant toxicological danger. *E. densa* does not have the ability to metabolize this compound (its concentration increased during the whole experiment); therefore, the level of chlorophyll detected within its tissue continued to decrease, which may cause necrosis in the plant.

## Electronic supplementary material

Below is the link to the electronic supplementary material.

ESM 1 (PDF 455 kb)

ESM 2 (PDF 260 kb)

## References

[CR1] Barker SA (2007). Matrix solid phase dispersion (MSPD). J Biochem Biophys Meth.

[CR2] Batisson I, Crouzet O, Besse-Hoggan P, Sancelme M, Mangot J-F, Mallet C, Bohatier J (2009). Isolation and characterization of mesotrione-degrading *Bacillus* sp. from soil. Environ Pollut.

[CR3] Bonnet JL, Bonnemoy F, Dusser M, Bohatier J (2008). Toxicity assessment of the herbicides sulcotrione and mesotrione toward two reference environmental microorganisms: *Tetrahymena pyriformis* and *Vibrio fischeri*. Arch Environ Contam Toxicol.

[CR4] Capriotti AL, Cavaliere C, Lagana A, Piovesana S, Samperi R (2013). Recent trends in matrix solid-phase dispersion. Trends Anal Chem.

[CR5] Casati P, Lara M, Andreo CS (2002). Regulation of enzymes involved in C4 photosynthesis and the antioxidant metabolism by UV-B radiation in *Egeria densa*, a submersed aquatic species. Photosynth Res.

[CR6] Chaabane H, Vulliet E, Jouxc F, Lantoine F, Conan P, Cooper J-F, Coste C-M (2007). Photodegradation of sulcotrione in various aquatic environments and toxicity of its photoproducts for some marine micro-organisms. Water Res.

[CR7] Csurhes S, Hannan-Jones M, Dimmock A (2008). Pest plant risk assessment: dense waterweed *Egeria densa*. Department of Primary Industries and.

[CR8] Dillon CR, Maurice DV, Jones JE (1988). Composition of *Egeria densa*. J Aquat Plant Manage.

[CR9] Favas PJC, Pratas J, Prasad MNV (2012). Accumulation of arsenic by aquatic plants in large-scale field conditions: opportunities for phytoremediation and bioindication. Sci Total Environ.

[CR10] Ferrat L, Pergent-Martini C, Roméo M (2003). Assessment of the use of biomarkers in aquatic plants for the evaluation of environmental quality: application to seagrasses. Aquat Toxicol.

[CR11] Hanson ML, Knapp CW, Graham DW (2006). Field assessment of oxytetracycline exposure to the freshwater macrophytes *Egeria densa* Planch. and *Ceratophyllum demersum* L. Environ Pollut.

[CR12] Jin B, Xie L, Guo Y, Pang G (2012). Multi-residue detection of pesticides in juice and fruit wine: a review of extraction and detection methods. Food Res Int.

[CR13] Knauer K, Hommen U (2013). Environmental quality standards for mixtures: a case study with a herbicide mixture tested in outdoor mesocosms. Ecotoxicol Environ Saf.

[CR14] Malec P, Maleva MG, Prasad MNV, Strzalka K (2009). Identification and characterization of Cd-induced peptides in *Egeria densa* (water weed): putative role in Cd detoxification. Aquat Toxicol.

[CR15] Marín VH, Tironi A, Delgado LE, Contreras M, Novoa F, Torres-Gómez M, Garreaud R, Vila I, Serey I (2009). On the sudden disappearance of *Egeria densa* from a Ramsar wetland site of Southern Chile: a climatic event trigger model. Eco Mod.

[CR16] Reid MA, Morin L, Downey PO, French K, Virtue JG (2009). Does invasive plant management aid the restoration of natural ecosystems?. Biol Conser.

[CR17] Riis T, Olesen B, Clayton JS, Lambertini C, Brix H, Sorrell BK (2012). Growth and morphology in relation to temperature and light availability during the establishment of three invasive aquatic plant species. Aquat Bot.

[CR18] Rodrigues SA, Caldas SS, Primelm EG (2010). A simple; efficient and environmentally friendly method for the extraction of pesticides from onion by matrix solid-phase dispersion with liquid chromatography–tandem mass spectrometric detection. Anal Chim Acta.

[CR19] Soltani N, Shropshire C, Sikkema PH (2011). Response of spring planted barley (*Hordeum vulgare L*.), oats (*Avena sativa L*) and wheat (*Triticum aestivum L*.) to mesotrione. Crop Prot.

[CR20] Su S, Zhou Z, Qin JG, Yao W, Ma Z (2010). Optimization of the method for chlorophyll extraction in aquatic plants. J Freshwat Ecol.

[CR21] Su S, Zhou Y, Qin JG, Wang W, Yao W, Song L (2012). Physiological responses of *Egeria densa* to high ammonium concentration and nitrogen deficiency. Chemosphere.

[CR22] US-EPA (2001) EPA pesticide fact sheet, 4.06.2001. US-EPA, Washington, DC

[CR23] Zhang L, Li S, Cui X, Pan C, Zhang A, Chen F (2012). A review of sample preparation methods for the pesticide residue analysis in foods. Cen Europ J Chem.

