# Supplementary material for: Extraction procedures for the study of phytotoxicity and degradation processes of selected triketones in a water ecosystem
Source: Environ Sci Pollut Res Int. 2013 Dec 21;21(6):4751–8. doi: 10.1007/s11356-013-2425-z (PMC3945236; doi:10.1007/s11356-013-2425-z)
Supplement: Supplementary file 1 — (PDF 455 kb) [file 11356_2013_2425_MOESM1_ESM.pdf]

Extraction procedures for the study of phytotoxicity and degradation processes of selected triketones in a water ecosystem

Environmental Science and Pollution Research

Hanna Barchanska*, Anna Kowalska, Barbara Poloczek

Department of Inorganic, Analytical Chemistry and Electrochemistry, Faculty of Chemistry, Silesian University of Technology, B. Krzywoustego 6 Str, 44 – 100 Gliwice, Poland

- - corresponding author, e – mail: hanna.barchanska@polsl.pl,

tel.: +48 32 2372818, fax.: +48 32 2371205,

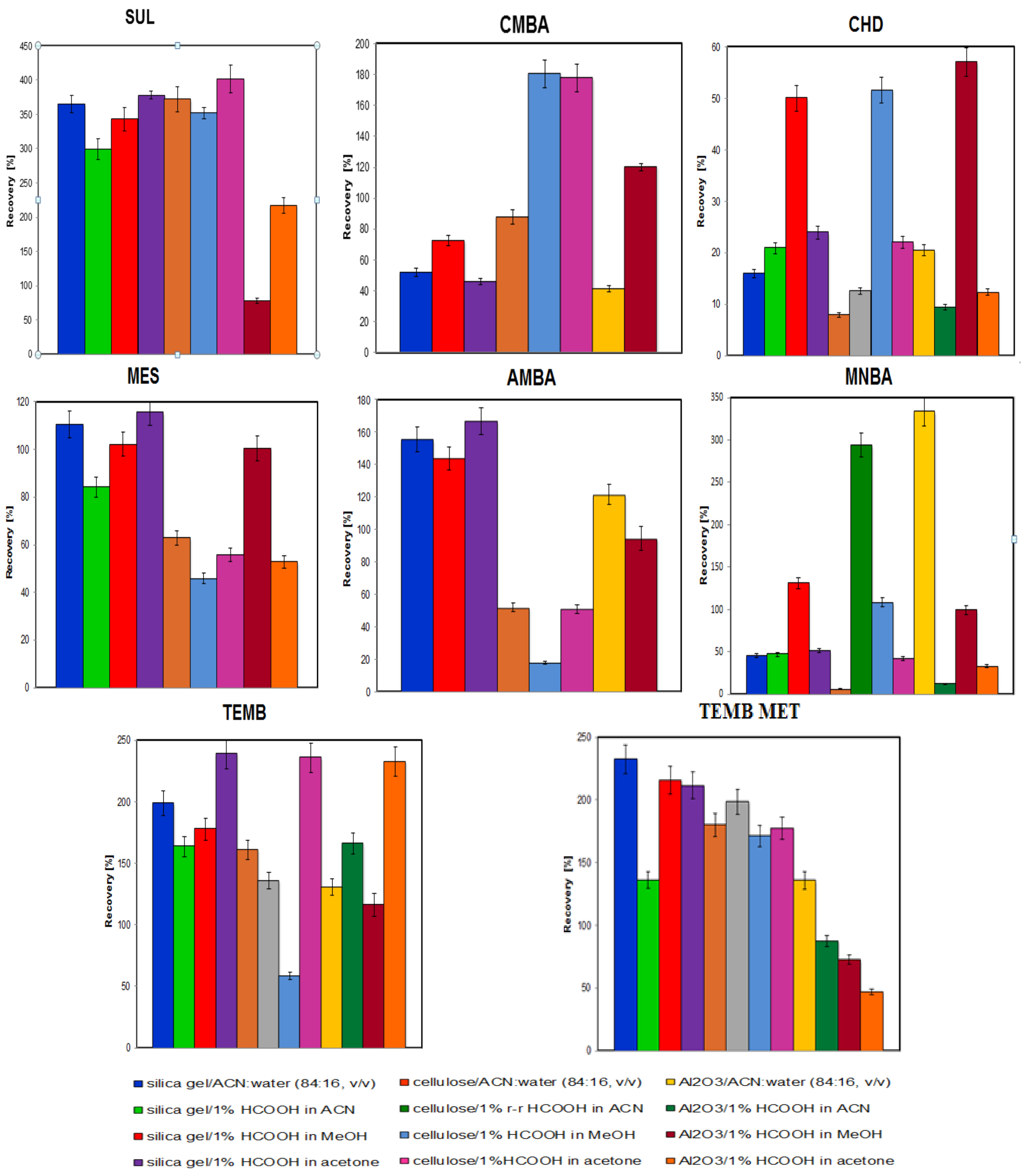

Fig.1 Analytes recovery from plant tissues obtained by MSPD procedure

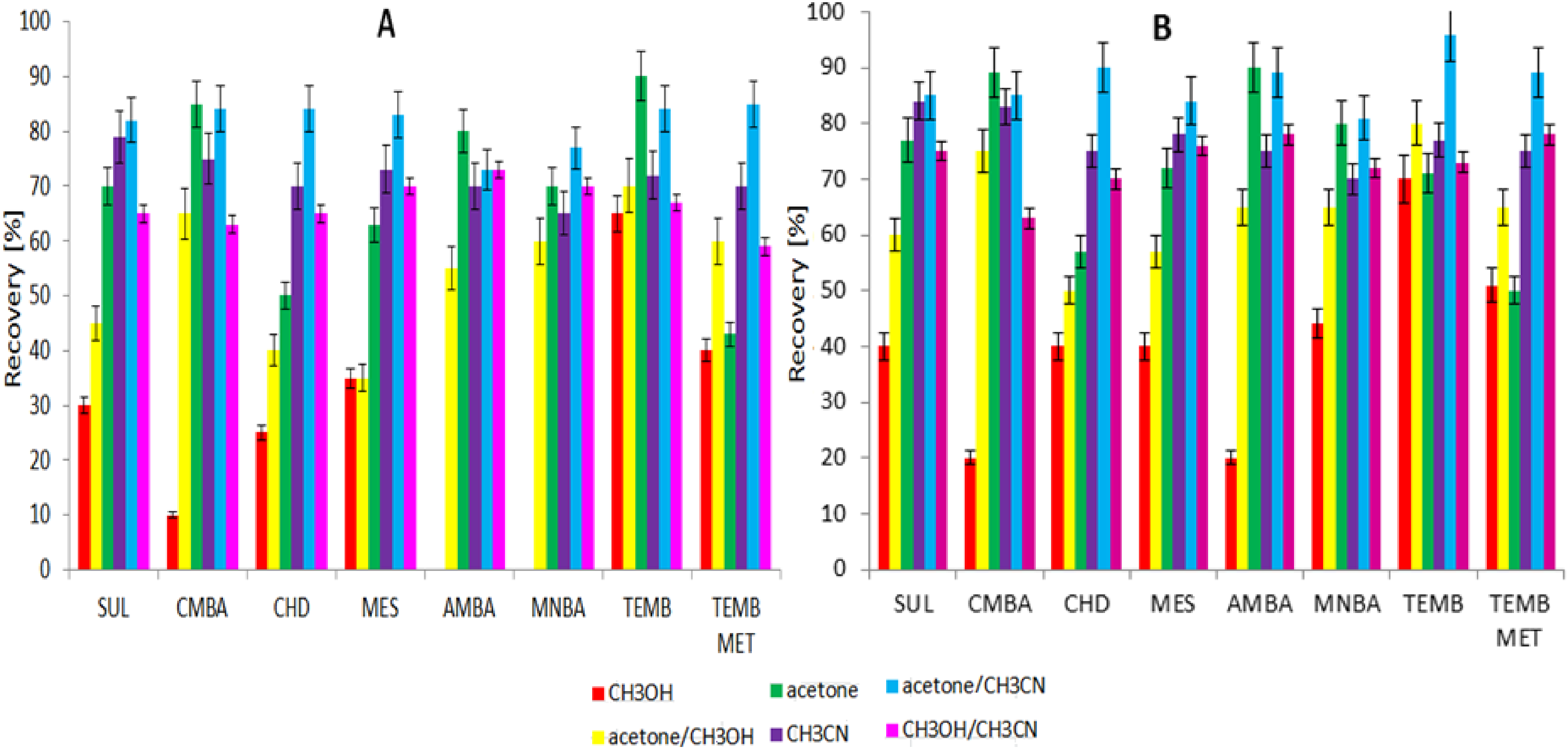

Fig.2 Recoveries of analytes from water samples obtained by DLLME, Fig.2A – extraction solvent: CCl_4; Fig.2B – extraction solvent: chloroform

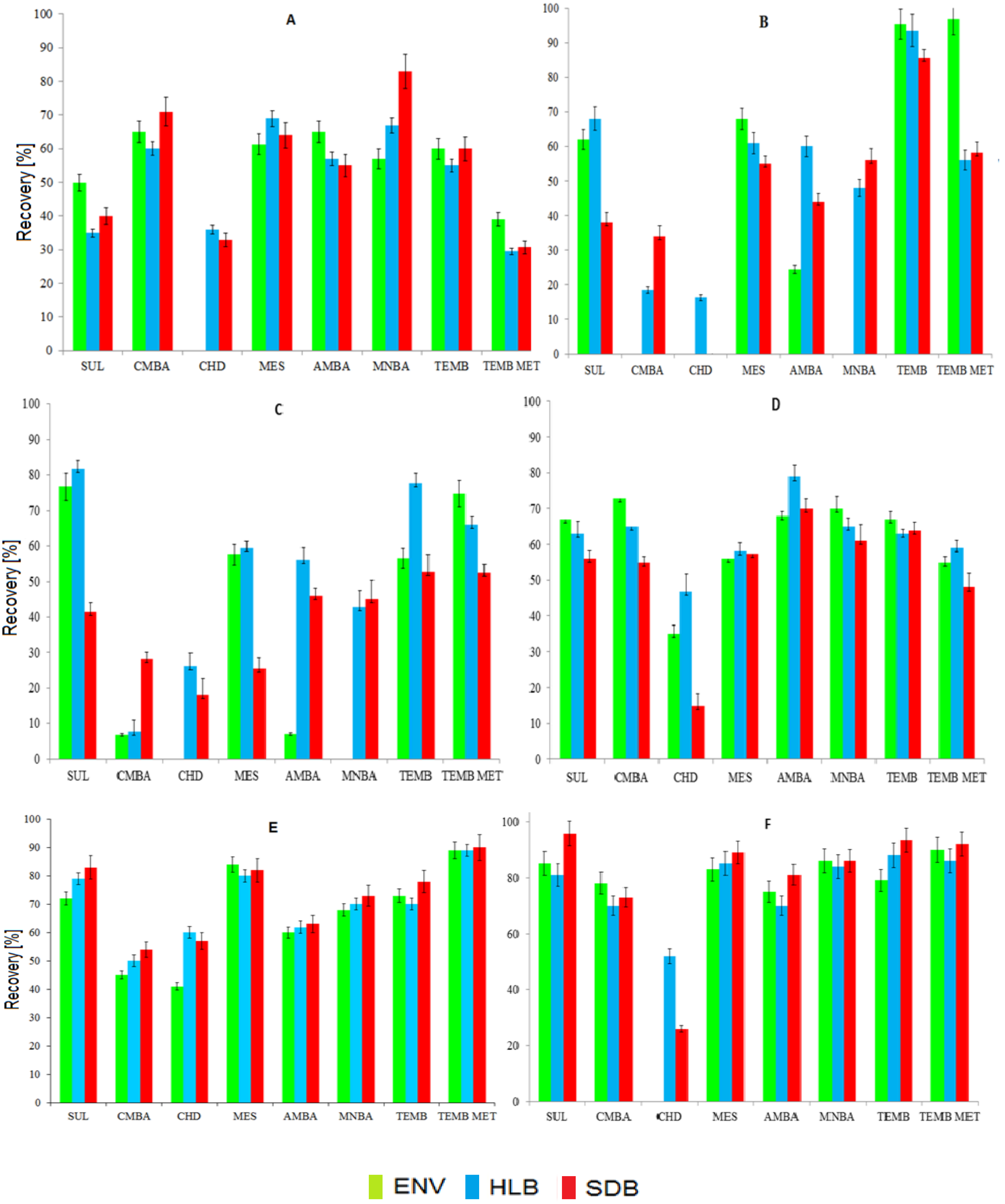

Fig.3 The recoveries of analytes from water samples obtained by SPE with different elution solvents: A – acetone, B – CH_3CN, C - CH_3OH, D – acetone/ CH_3OH, E – acetone/CH_3CN, F - CH_3OH/CH_3CN
