# Supplementary material for: Extraction procedures for the study of phytotoxicity and degradation processes of selected triketones in a water ecosystem
Source: Environ Sci Pollut Res Int. 2013 Dec 21;21(6):4751–8. doi: 10.1007/s11356-013-2425-z (PMC3945236; doi:10.1007/s11356-013-2425-z)
Supplement: Supplementary file 2 — (PDF 260 kb) [file 11356_2013_2425_MOESM2_ESM.pdf]

Extraction procedures for the study of phytotoxicity and degradation processes of selected triketones in a water ecosystem

Environmental Science and Pollution Research

Hanna Barchanska*, Anna Kowalska, Barbara Poloczek

Department of Inorganic, Analytical Chemistry and Electrochemistry, Faculty of Chemistry, Silesian University of Technology, B. Krzywoustego 6 Str, 44 – 100 Gliwice, Poland

- - corresponding author, e – mail: hanna.barchanska@polsl.pl,

tel.: +48 32 2372818, fax.: +48 32 2371205,

1. *Optimalization of the MSPD procedure*

For the recovery study, waterweed with target pesticides non-detected was used as alternative matrices and spiked at 5 μg/g to determine method recoveries. Most of investigated compounds were polar (Table 1), and therefore for their isolation from plant matrix polar sorbents such as silica gel, aluminum oxide and cellulose were chosen. The analytes elution was conducted by means of 1% HCOOH in acetone, 1% HCOOH in methanol, 1% HCOOH in acetonitrile, and the mixture acetonitrile-water (85:15, v/v). After optimization, the proportion 0.5 g of plant tissues to 1.0 g of sorbent, and the elution of analytes with 4 mL of solvent were established as optimal for these analytes.

The mean recoveries of analytes obtained under different extraction conditions with error bars are shown in Figure 1.

Fig.1 Analytes recovery from plant tissues obtained by MSPD procedure

A possible cause for analyte recoveries over 100% is the interference of matrix compounds. Although *Egeria densa* tissues contain 90–95% water, other components, primarily proteins (13% of dry mass), and non-polar compounds (10% of dry mass), as well as lignin and cellulose (26% of dry mass) (Dillon et al. 1988), hamper the chromatographic analysis.

2. Optimization of the DLLME procedure

Acetone, acetonitrile, methanol and their mixtures in proportion 1:1 (v/v) were selected as disperser solvents. All of them have unlimited water solubility and logP below zero.

As the extraction solvents, chloroform and carbon tetrachloride were used, whereas as disperser acetone, acetonitrile, methanol, and mixtures of these solvents were applied. The lowest values were obtained for methanol as disperser solvent, regardless of extraction solvent, which is related to the lowest value of logP (-0.74) among the tested solvents. Acetone gave very high recoveries (80-90%) of semi–polar compounds CMBA, AMBA and TEMB, but the recoveries of the polar analytes CHD and TEMB MET were below 60%. Polar analytes, especially those with logP < 1 are more soluble in the mixture of water and methanol. Therefore, the recovery of these analytes was low when methanol (pure or in a mixture) was applied as a disperser. As an extraction solvent, chloroform was chosen. It provided higher recoveries of all analytes in comparison to carbon tetrachloride and its higher density provided easier phase separation. The detailed data are presented in Figure 2.

Fig.2 Recoveries of analytes from water samples obtained by DLLME, Fig.2A – extraction solvent: CCl_4; Fig.2B – extraction solvent: chloroform

3. Optimization of the SPE procedure

SPE, a well-established technique, was employed for the verification of the results obtained by DLLME. Analyte-free samples were spiked with standards solution to obtain two concentration levels (10 μg/L and 100 μg/L) to determine method recoveries. The sample volume was 250 mL. Three different sorbents (ENV, HLB, and SDB) were tested. In all cases, sorbents conditioning was performed by means of 6 mL of methanol, and subsequently 6 mL of water. The analytes elution was conducted by means of 6 mL of acetone, acetonitrile, methanol, and the mixtures of above solvents (in proportion 1:1, v/v). After analytes elution, the extract was evaporated to dryness under a stream of nitrogen and the residue was dissolved in 1 mL of methanol. The analytes recoveries are presented in Figure 3.

Fig.3 The recoveries of analytes from water samples obtained by SPE with different elution solvents: A – acetone, B – CH_3CN, C - CH_3OH, D – acetone/ CH_3OH, E – acetone/CH_3CN, F - CH_3OH/CH_3CN

Acetone, methanol, and acetonitrile applied individually as elution solvents provided low recoveries (rarely exceeding 70%), irrespective of the type of sorbent. Also, the mixture acetone/methanol did not provide satisfactory recoveries (15-78%). Under the above mentioned conditions, the lowest recovery (15%) was obtained for 1,3-cyclohexanedione (CHD). Its structure is different from the other compounds. CHD has the same value of logP as MES (logP = -0.99), but its chemical structure and pK_a values (5.26) mean that it is poorly retained on semi-polar sorbents. The highest retention and thereby the recovery of CHD (48%) **w**as achieved on HLB sorbents.

Application of the mixtures of acetone, methanol, and acetonitrile for analytes elution led to the improvement of the extraction efficiency. The acetonitrile/methanol mixture provided the highest efficiency of elution of triketones and their degradation products from the tested sorbents.

For triketones determination in real water samples, the HLB sorbent was applied and the analytes were eluted with the mixture of acetonitrile/methanol (1:1, v/v). This procedure provided the analytes recovery in the 52–range of 88% range.
